# A Comprehensive Review on Crustaceans’ Immune System With a Focus on Freshwater Crayfish in Relation to Crayfish Plague Disease

**DOI:** 10.3389/fimmu.2021.667787

**Published:** 2021-05-13

**Authors:** Younes Bouallegui

**Affiliations:** LR01ES14 Laboratory of Environmental Biomonitoring, Faculty of Sciences of Bizerte, University of Carthage, Bizerte, Tunisia

**Keywords:** hemocytes, hyaline cells, hematopoiesis, etosis, *Aphanomyces astaci*

## Abstract

Freshwater crayfish immunity has received great attention due to the need for urgent conservation. This concern has increased the understanding of the cellular and humoral defense systems, although the regulatory mechanisms involved in these processes need updating. There are, however, aspects of the immune response that require clarification and integration. The particular issues addressed in this review include an overall description of the oomycete *Aphanomyces astaci*, the causative agent of the pandemic plague disease, which affects freshwater crayfish, and an overview of crustaceans’ immunity with a focus on freshwater crayfish. It includes a classification system of hemocyte sub-types, the molecular factors involved in hematopoiesis and the differential role of the hemocyte subpopulations in cell-mediated responses, including hemocyte infiltration, inflammation, encapsulation and the link with the extracellular trap cell death pathway (ETosis). In addition, other topics discussed include the identity and functions of hyaline cells, the generation of neoplasia, and the emerging topic of the role of sessile hemocytes in peripheral immunity. Finally, attention is paid to the molecular execution of the immune response, from recognition by the pattern recognition receptors (PRRs), the role of the signaling network in propagating and maintaining the immune signals, to the effector elements such as the putative function of the Down syndrome adhesion molecules (Dscam) in innate immune memory.

## Introduction

The large decline in the number of the European indigenous freshwater crayfish caused by repetitive outbreaks has brought such organisms close to extinction (e.g., *Astacus astacus*) (1–3). Scientists have raised their concerns about the natural stock of endangered crayfish species, boosting investigations to uncover the causes for conservation purposes. The crayfish plague disease, reported as the main threat to this species is linked to the accidental or commercial relocation of invasive species like the North American species (e.g., *Pacifastacus leniusculus* and *Procambarus clarkii), a* vector of the oomycete *Aphanomyces astaci*, the causative agent of the disease (4–6). The disease’s most characteristic symptoms are common spots of melanin in the cuticle of infected or dead individuals. Extensive studies have demonstrated that such melanin spots (i.e., melanization) are the result of the immune response aiming to enclose the pathogen in melanin sheaths to prevent it from invading and grow into the hemocoel (7–11). *A. astaci* is capable of infecting indigenous and invasive crayfish species equally, however, the latter are less vulnerable (*P. leniusculus, C. quadricarinatus, and Procambarus clarkii*) (1, 8, 12). However, vulnerable indigenous European species like *A. astacus*, mostly showed very few or almost no formation of melanized spots, while the development of hyphae was recorded. The differential immune response between indigenous and invasive species is among the main clues that need to be studied and has driven investigations related to crayfish immunity (4, 13, 14). In this context, the activation of the prophenoloxidase (ProPO) system, the process responsible for melanin formation, has been extensively studied (15–21). Interestingly, previous studies have demonstrated that encapsulation is a mandatory process that triggers the activation of the ProPO system (22–24). However, functional mechanisms linking cooperative actions of the humoral- and cell-mediated responses of the innate immunity, need to be considered further (18, 25). In doing so, the relationships between melanization, pathogen encapsulation, and immune cell production (i.e., hematopoiesis) need to be elucidated and integrated to the reported decrease in the number of circulating hemocytes (i.e., total hemocyte count [THC]), during infection (14–17, 26–31). In this context, the immune modulation of freshwater crayfish against *A. astaci* is still hampered by a smaller integration of the components of functional defenses (17, 32). In this review we tried to compile different components of the crayfish immune response, related strategies, and debated topics in one paper, aiming to make a comprehensive and integrated synopsis of the crayfish immune system available that might advance the understanding of this topic.

## *Aphanomyces astaci*: causative agent of the Crayfish Plague Disease

The pathogen *Aphanomyces astaci* (Shikora, 1906) is a pathogenic oomycete native to North America and only colonizes aquatic decapods. It is listed among the world’s 100 worst invasive alien species (3, 33). The oomycetes, the so-called water molds, belong to the genus *Aphanomyces* and are members of the *Ooomycotina*, *Eumycota* family, which are not true fungi but more closely related to brown algae and diatoms (8, 33). *A. astaci* is a non-septate, branching fungus belonging to the *Saprolegniaceae* family, with 7-10 micron thick hyphae and biflagellate zoospores assuring their asexual reproduction. The life cycle comprises a relatively short-lived swimming zoospore stage that ends with encasement (12). Encysted spores drop or retract their flagella to be able to encase into the cell wall covered by sticky substances assuring their adherence to the host. Shortly after, a period of hyphal growth within the host starts and the emerging hyphae penetrate the host’s cuticle (8, 12). So far, 5 strains of *A. astaci* have been identified and characterized to belong to 5 different genotypes thanks to random amplified polymorphic DNA (RAPD) (2, 34, 35). Such genotypes are referred to as groups 1, 2, 3, and 4, or as the *Astacus* strain, the *Pacifastacus* strain I, the *Pacifastacus* strain II, and the *Procambarus* strain, which can be abbreviated to As, PsI, PsII, Pc, and Or, respectively. The As-strain is referred to as *Astacus*, and is the first genus from which this strain was identified since the original host has not yet been identified or found. The original host of PsI and PsII is the crayfish species *Pacifastacus leniusculus* (Dana, 1852), the original hosts of the Pc and Or genotypes are *Procambarus clarkii* (Girard, 1852), and *Orconectes limosus* (Rafinesque, 1817), respectively (2, 10, 34, 36, 37). The As-genotype was thought to be responsible for the first outbreaks in Europe following its transmission in the 19^th^ century. In contrast to North American crayfish species, which maintain a relatively stable interaction with *A. astaci*, Asian, Australian, and European crayfish species are highly susceptible to the pathogen. However, recent reports have recorded differential virulence of different *A. astaci* strains to different European species (36, 37). Laboratory experiments demonstrated that PsI was found to be the most virulent causing all the noble crayfish *Astacus astacus* to die within few days, while the As strain was found to be the less virulent in general (36). Overall, a devastating impact on European-native crayfish species has been recorded, which drastically declined their natural stock and so far has led to numerous species being listed at “risk of extinction” (34). Many studies have attributed this drastic effect to the inability of such species to respond efficiently to prevent the spread of *A. astaci*. Thus, the oomycete is capable of killing almost 100% of the individuals within an infected population in a few days (8, 10, 12). Infected individuals of vulnerable species are incapable of preventing hyphae growth in their hemocoel, in contrast to resistant species, which have been reported to extensively respond by triggering their immune system after interaction with the oomycete cell wall (1,3-β-glucan). This stimulates the activation of the prophenoloxidase system producing cytotoxic compounds and melanin to ensure the encapsulation of the pathogen in the cuticle and prevent it from completely colonizing the hemocoel (8, 34). There is no efficient treatment for the disease, nor has there been a successful strategy recorded to help mitigate the pathogen’s effects. However, a deep understanding of defense mechanisms, which has greatly increased nowadays, is still the most promising way to design an accurate strategy to overcome this threatening situation.

## Epidemiology and Prevalence of the Pathogen *Aphanomyces astaci*

*Aphanomyces astaci* outside of North America is considered as an alien species. The first evidence for its introduction into Europe marked by the first mass mortality among European native crayfishes occurred in 1859 in the Po River basin in northern Italy, which has deeply threatened populations of the Italian white-clawed crayfish *Austropotamobius pallipes* (38). These followed later by several outbreaks in the Plateau des Langres at the Franco-German frontier area, in 1874 (38). The following decades, witnessed steady spreads of the pathogen to other European countries following main two directions, down the Danube river into the Balkans ending in the Black Sea, and across North Germany into Russia. From there, the distribution occurred toward the south again to the Black Sea, and onto north-west to Finland and, in 1907, to Sweden. And since, that time it has spread across most of Europe. However, in 1960s, the first pandemics were reported in Spain, and two decades later the disease spread far to the British Isles, Turkey, Greece and Norway (39). Currently, the distribution of *A. astaci*, is confirmed to be present in other continents rather than native, and classic localities across Europe, like South America, Japan (40). Introductions of invasive crayfish species in many sites around the world for aquaculture purposes have been usually found to be coupled by A. astaci. However, no further data has been recorded about the distribution of A. astaci in Africa, Central America, South America, and other parts of east and south Asia (40). Australia and New Zealand have not experienced any outbreaks of crayfish plague to date and are currently considered plague free area (40). The actual distribution of A. astaci is therefore likely to be far broader than the distribution table would suggest.

Very recently, the occurrence of chronic infections in native European crayfish populations were documented, however such phenomenon still newly reported, and underlying mechanisms and factors remain scarce and need to be investigated further (1, 11, 37). Recent assessments on the potential of freshwater crabs, and shrimp (11, 37, 41) to be as alternative hosts have been conducted to rule out non-lethal effects in the absence of any mass mortality. The following is an integrated illustration of the recent findings on the crayfish immune system.

## General Description of Crayfish Immunity

**Figure 1:**
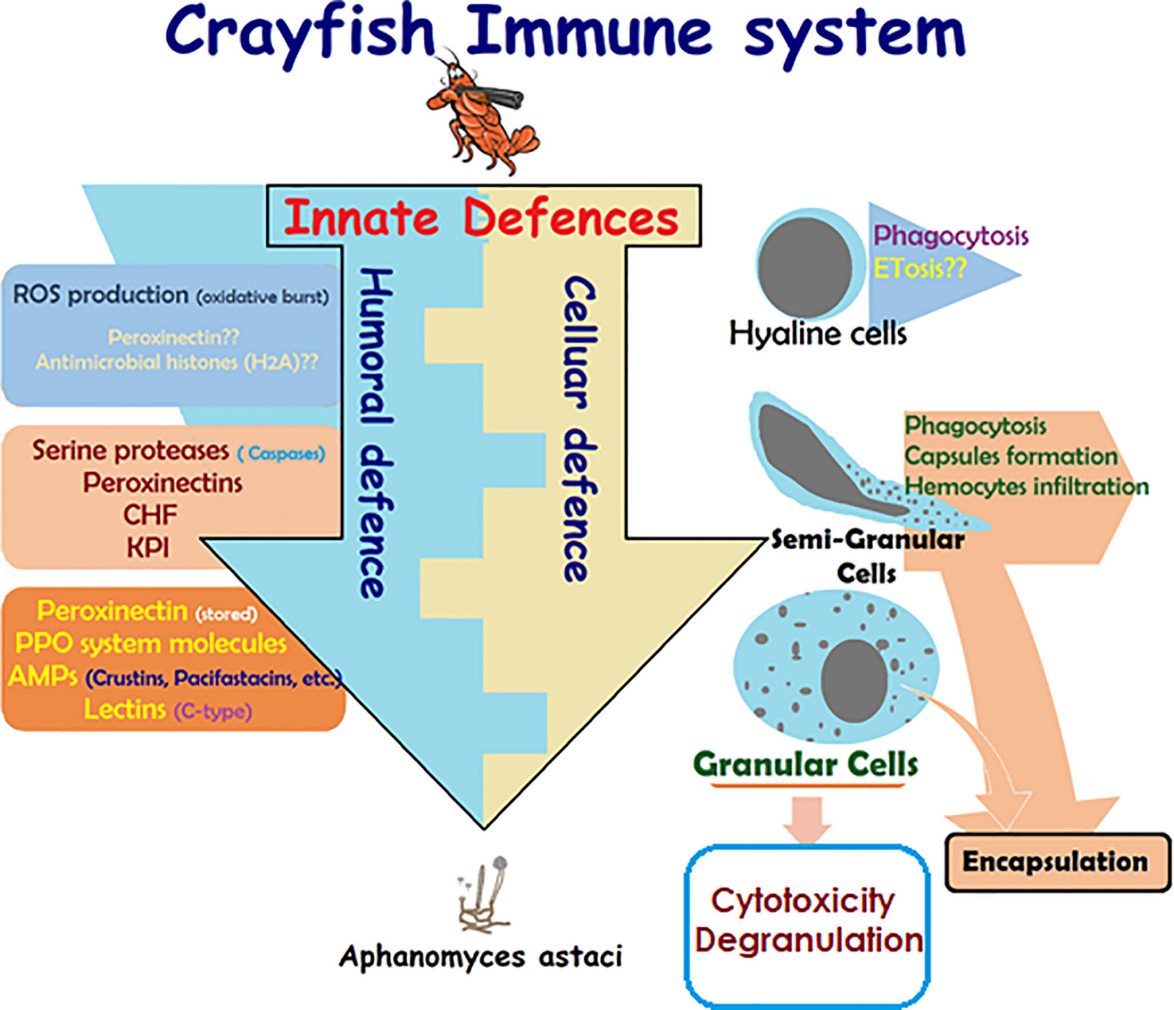
Schematic presentation of crayfish immune response. Presentation of the main constituents of crayfish immune response alongside the cooperative action of the cellular-mediated and humoral-mediated responses and their corresponding factors (4, 5, 14, 17, 18, 26, 28–30, 42–54).

Like all invertebrates, crustaceans, including crayfish, only rely on innate immunity, which constitutes humoral- and cellular-mediated responses (**Figure 1**) (17, 18, 29, 30, 42). Lack of acquired (specific) immune response in crayfish is due to the lack of myeloid lineage cells being able to produce immunoglobulins (specific antibodies) against specific pathogens in the event of repetitive infections (lack of memory). This lack of acquired immune response was found to be balanced by the enrichment of different cell-mediated strategies and several multi-type humoral factors, both constituting the innate defenses of the crayfish immunity (**Figure 1**) [e.g., multiple pathogen recognition receptors (PRRs)] (28, 42). These defenses are activated in a cooperative way in order to initiate effective defensive actions and can be classified according to two main responses: a humoral-mediated response involving humoral factors produced and released by the immune cells (antimicrobial peptides, hydrolytic enzymes, and molecules of the ProPO system, etc.), and a cell-mediated response that requires a direct action of the cell itself, such as phagocytosis, initiating the cell death pathway mechanisms (e.g., cytotoxicity, encapsulation) (**Figure 1**).

### Cell-Mediated Immune Response

Cell-mediated responses require the direct action of the cell itself to neutralize alien invaders through mechanisms like phagocytosis, cell death pathways (e.g., apoptosis and autophagy), and inflammatory response (e.g., nodule formation and encapsulation). These strategies share a common prerequisite, which is hemocyte infiltration (hemocytosis), or the excessive migration of hemocytes from circulation to the infection site.

Changes in hemocytes’ shape and/or features, in addition to surveying the total hemocyte number (THC), and differential hemocyte count (DHC), as well as the assessment of defensive activities like phagocytosis, cell viability, cytotoxicity, and different physiological changes, have been considered useful assays to investigate the crayfish immune response modulation (4, 26, 43–45). Hemocytosis requires extensive recruitment and infiltration of hemocytes into tissues where infections/stress have been detected (25, 30, 32, 55, 56). Hemocyte infiltration may result in structural modifications of the histology of target tissue near the infection-surrounding microenvironment (56–66). Inflammatory response and histopathological assessments linked to hemocyte infiltration have been integrated into a single quantitative index using multiple histopathological traits for a more sensitive discrimination of the health status of assessed organisms like bivalves and fish aiming to develop an accurate system for assessing the health status of farmed animals (67–70). However, very few studies have been conducted on crayfish histopathology. Interestingly, studies using hemocyte parameters as a tool for assessing the modulation of the immune response in crustacean (mainly THC and DHC describing the fall in the number of circulating hemocytes due to their recruitment to infected tissues, to block pathogens from disseminating the hemocel), could be inter-correlated with quantitative histopathological assessments for more accurate hemocyte parameter data and integrative conclusions (4, 14, 15, 18, 24, 26).

It is worth noting that the activation of melanin production by the ProPO system in crustacean defenses might be disrupted due to a lack of the appropriate number of hemocytes needed to fulfill such a role. This finding has been highlighted in *Astacus astacus* infected with *Aphanomyces astaci*, and has been linked to the lack of melanization in infected tissues that present hyphae development, which is not the case in invasive species like *Pacifastacus leniusculus* (species showing resistance to *A. astaci*) (4, 5, 46). In this context, it would be interesting to illustrate the characteristics of hemocytes (and their different sub-populations), the key element of the crayfish immune response, which will be detailed in the following sections.

## Classification of Hemocyte Sub-populations

### Morphological Characterization/Classification

Hemocytes, the key element of the crayfish immunity, participate in almost every defensive activity through direct action (e.g., phagocytosis, capsule formation), in addition to their production of most humoral factors (17). Hemocytes have been described and characterized since the early 1880s by Carus as reported in (47). Hemocyte sub-populations were classified in general following morphological characterization according to the size of the cell, and the structural complexity of their cytoplasm (i.e., nucleus/cytoplasm ratio and the presence/absence of granules) (15, 24, 26, 71). Such characterization was mostly consolidated by a functional/cytochemical classification of each sub-population (i.e., dependent on the main defensive action and/or enzymatic activity through their enclosed granules, like peroxidase) (72–74). Last but not least, despite the significant progress recorded in these studies, the final number of crayfish hemocyte subpopulations has not yet been firmly determined. However, the oldest and most accepted clustering system presents three hemocyte sub-types: hyaline cells (HC) also called hyalinocytes, semi granular cells or hemocytes (SGC), and granular cells or hemocytes (GC), also called granulocytes (26, 48, 49). The general characteristics are summarized in **Table 1** below. Hyaline cells (HC) are described as the smallest cell type, while SGC typically feature an elongated shape. On the other hand, GC is described as the largest cell-type (43–46, 50, 51).

**Table 1:** Summary of the three hemocyte sub-populations’ characteristics.

Hemocyte sub-populations	Size (mean Ø)	Characteristics(structural complexity of cytoplasm)
**Hyaline cells** **(HC)**	10 µm	• Oval-shaped cells • High nucleus-to-cytoplasm ratio • Euchromatic centered nucleus • Very few granules
**Semi-granular cells** **(SGC)**	15 µm	• Low nucleus-to-cytoplasm ratio • Elongated cells • Large central nucleus • Well-developed organelles (rough endoplasmic reticulum) • Numerous granules
**Granular cells** **(GC)**	21 µm	• Very low nucleus-to-cytoplasm ratio • Spindle-shaped cells • Enclosed nucleus; finely dispersed nucleoplasm • Large, well developed, structureless lemon-like granules

### Functional Characterization

Functional activities of each hemocyte sub-type still are still the cause of some controversy (5, 14). This classification started to be elucidated by the early 1980s, however, separation techniques developed by Smith and Söderhäll at that time (1983) greatly advanced the detailed description of said functional activities (26). In this context, hyaline cells were described as phagocytic cells, semi granular cells were the main type ensuring encapsulation, and granular cells were mainly specialized in storing/releasing active molecules such as prophenoloxidase (pro-PO) system molecules within/from their well-developed granules (14, 15, 18, 24, 26, 27). However, all the sub-types mentioned were reported to be involved in the immune response with differential efficiencies toward mechanistic processes (18, 43, 45, 52–54). For example, for the most studied crayfish species (e.g., *Astacus astacus, Pacifastacus leniusculus*, *Cherax quadricarinatus*, and *Procambarus clarkii*), semi granular cells are described as the most important family in immune reactivity, involving capsule formation, encapsulation and assuring moderate phagocytic activity in addition to melanization through activation of ProPO cascading reactions (4, 14, 43, 45). ProPO activation is the main role of granular cells that do not present any phagocytic activity. The phagocytic function has been mainly allocated to hyaline cells (**Figure 1**). Unfortunately, many investigations (*in vitro* and *in vivo*) reported controversial data about the functional activity of hyaline cells (43, 45) and this is due to their very small presence in circulation. The scarce presence/putative function of hyaline cells and their importance has been exacerbated by the lineage paradigm of different types of immune cells, which consists of immune cell proliferation exclusively occurs in the hematopoietic tissue, therefore only very well differentiated and mature cells are released into circulation. Mature semi-granular as well as granular cells cannot be fully activated until released into circulation (47, 53, 75). Unfortunately, HC are cells with the characteristics of immature undifferentiated cells (presenting proliferative phenotype, expressing PCNA; commonly called pro-hemocytes). HC, have been described to be released under certain circumstances, mainly after an immune challenge (46, 47) and so they have drawn attention to the existence and functions of such cell types (detailed in the following sections).

### Molecular Markers of Different Hemocyte Sub-populations

Further, many transcriptomic and proteomic studies have helped classify hemocyte cell-types, following specific molecular markers expressed by each type, and have improved their functional characterization (5, 46, 76). The PIRunt transcription factor has been reported as not being expressed in less mature cells, whereas it is marked simultaneously with the ProPO in the SGC and CG. More extension of ProPO to GC has been demonstrated. Furthermore, immunohistochemical studies reported the presence of superoxide dismutase (SOD) in SGC and GC, however, mRNA expression studies demonstrated that GC produces SOD (as monomers), which attaches to the outside of SGC (dimeric). More specifically, a Kazal-type proteinase inhibitor (KPI) and crustacean hematopoietic factor (CHF) were reported as being exclusively expressed by SGC (more details can be found in 5, 73, 62, 76). Very recent studies have shown that hemocytes of the freshwater crayfish *P. leniusculus* express two types of transglutaminases, commonly named Pl_TGase1 and Pl_TGase2, and have evidenced that such TGs are specific to different hemocyte sub-populations (77). Pl_TGase1 was only expressed in SGC, but not expressed by all SGCs, and Pl_TGase2 was only expressed in GC, and again not expressed by all GCs (77).

It’s worth to note that benefit from advanced single-cell RNA-sequencing (scRNAseq) technologies, in recent years, provide substantial improvement in reporting the hemocyte sub-type of the fly Drosophila, which has been largely renewed from only three sub-types: plasmatocytes, crystal cells, and lamellocytes to generating a comprehensive gene expression profiles for the drosophila circulating’ hemocytes. In fact, Fu et al. have identified two new hemocytes’ sub-types in Drosophila: thanacytes and primocytes with differential sets of genes (78). However, thanacytes expresses genes involved in distinct responses to different types of bacteria, while primocytes expresses cell fate, and signaling genes, potentially involved in keeping stem cells in the circulating blood. In addition to four other novel plasmatocytes subtypes (Ppnþ, CAH7þ, Lspþ and reservoir plasmatocytes), each with unique molecular markers and distinct predicted functions (78). Further study conducted by Tattikota et al., aimed to use scRNAseq in mapping hemocytes across different inflammatory conditions in larvae. Subsequently, different genes expression-based states of plasmocytes have been resolved (79). In addition, a rare subsets of crystal cells expressing the fibroblast growth factor (FGF) ligand branchless, and lamellocytes expressing receptor breathless, have been reported. FGF components are required for mediating effective immune responses against parasitoid wasp eggs. As perspective, scRNAseq analysis consists a very urgent needed analysis that should be performed to decipher the gene expression profiles for a systems-level understanding of the diversity of crayfish hemocytes, and their functions.

### Production of Hemocyte Sub-populations: The Hematopoiesis

**Figure 2:**
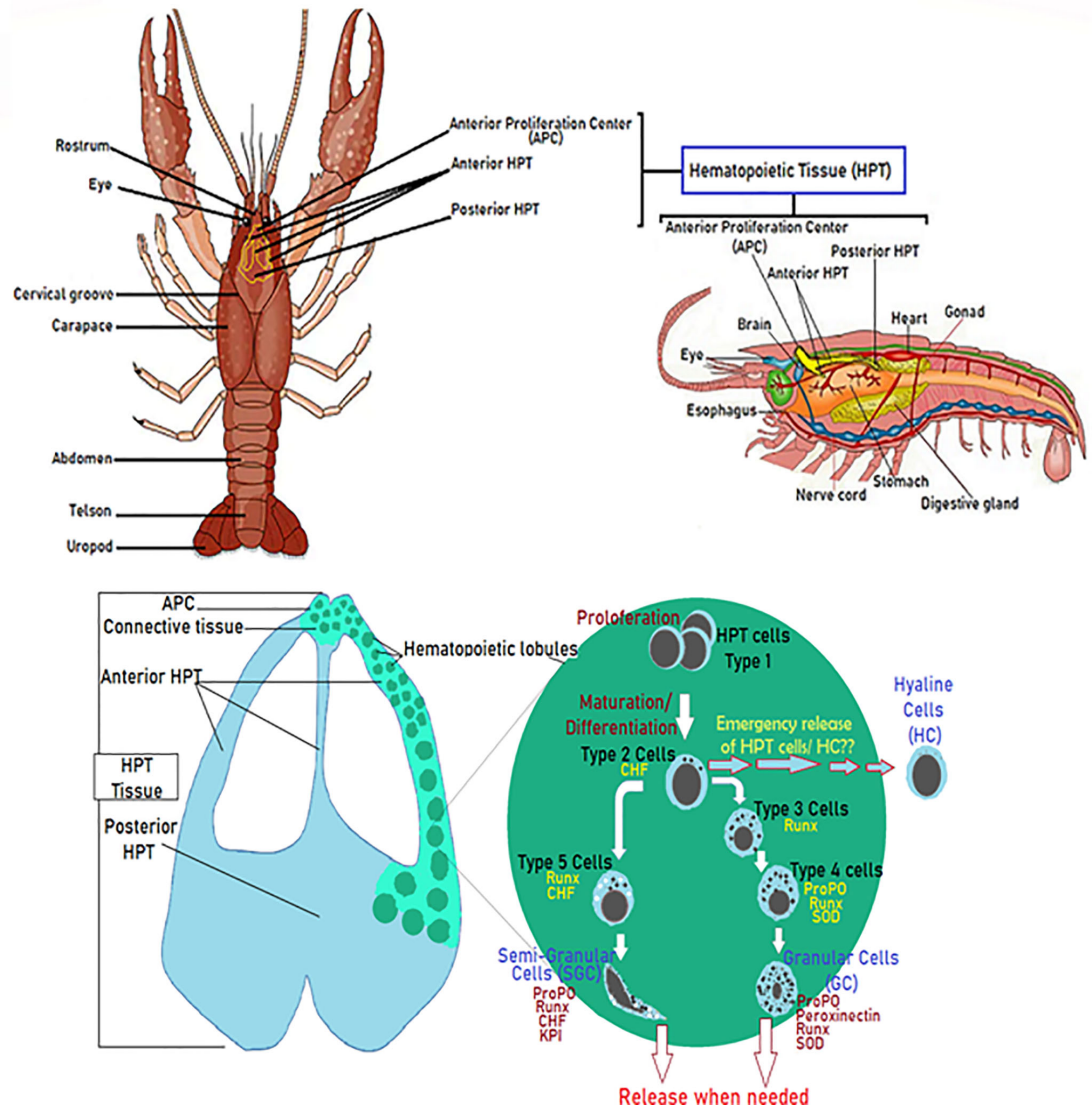
Diagram of the hematopoietic tissue (HPT) illustrating the hematopoiesis’ mechanistic steps. Schematic localization and presentation of the different parts of the HPT and the hematopoietic lobules illustrating the different phases of hemocyte production and how different cell types (Type I-V) acquire their specific molecular markers (47, 53, 75, 80–84).

Hematopoietic processes are among the most studied and have been really well described in studies related to crayfish immunity, aiming to decipher mechanisms underlying insufficient immune response against diseases like the plague disease (75, 80–82). Overall, investigations conducted by Söderhälls (Kenneth and Irene) and coworkers, resulted in an almost complete overview of molecular mechanisms that generate different hemocyte sub-families (47, 53, 82). In brief, the hematopoietic tissue in *P. leniusculus* contains four to five cell lineages arranged in lobules surrounded by connective tissue localized starting around the brain and following the ophthalmic artery to the posterior HPT, and ending by covering the dorsal part of the stomach (**Figure 2**) (47, 53). HPT cells are clustered into lobules of 10 cell clusters for each lobule (47, 75, 80, 83, 84). Cell morphology within such lobules is similar to different areas of the posterior HPT, with low mitotic activity in the center tissue that surrounds the ophthalmic artery. In contrast, high mitotic activity has been detected along the edges of HPT and the anterior proliferation center (APC), (**Figure 2**) (47, 75, 83). HPT cells clustered into as type I to type V cells, with type I have the characteristics of undifferentiated cells without or with few granules (high mitotic figures and expression of PCNA), located in the apical part of the lobule where proliferation (extensive division) of precursor/stem cell occurs. Tending towards the center of the lobule, cells being more determined, and differentiated. Type II-IV cells are distally arranged starting with the type II cells, all presenting granules. Type III and IV found between lobules separated from each other, do not show divisive activities and ready to be easily freed as mature hemocytes into circulation (47, 82). Type V showed granules morphologically distinct from other types (**Figure 2**) (47, 75, 80, 82, 83).

**Figure 3:**
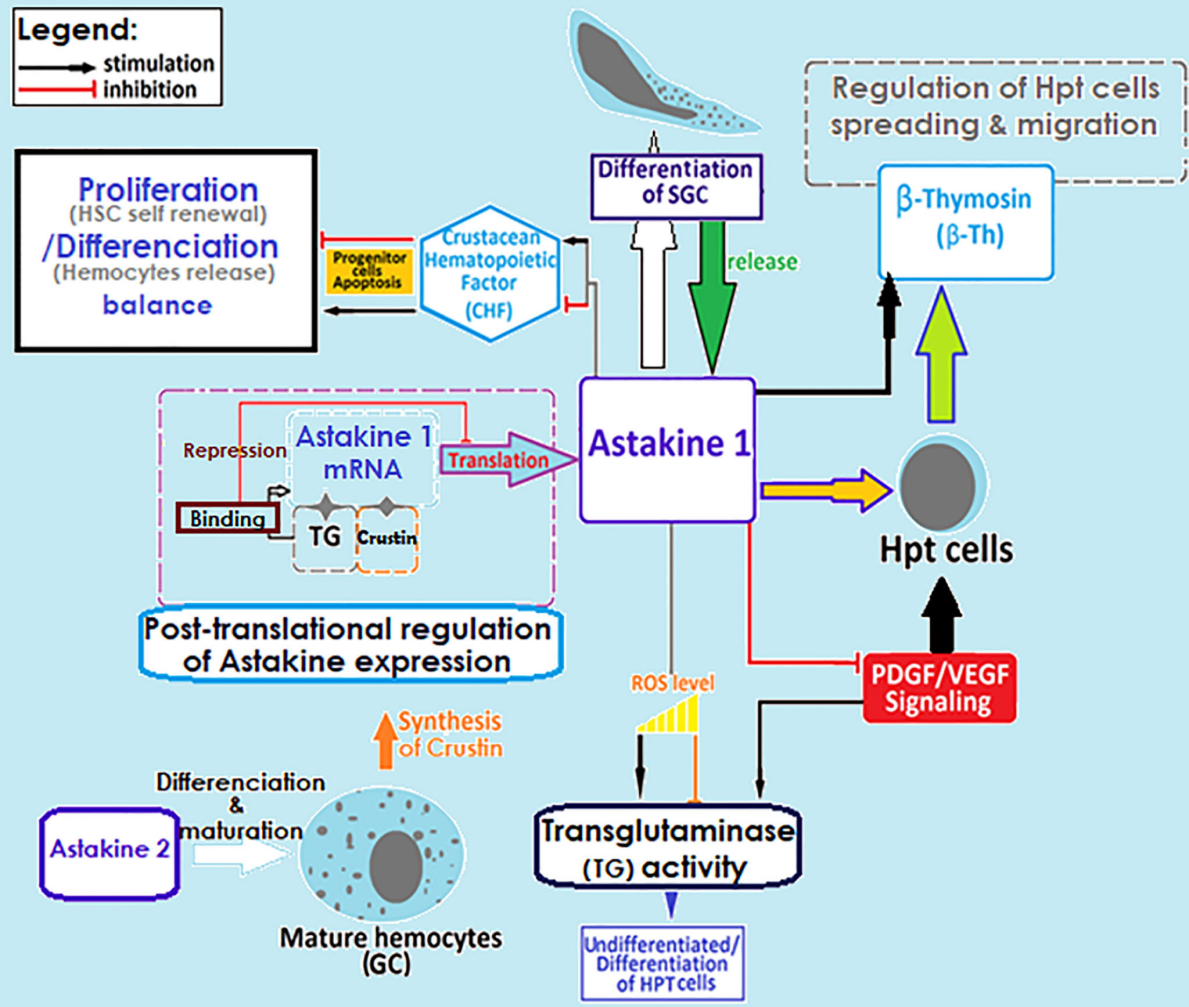
Regulation of crayfish hematopoietic mechanisms. Molecular factors involved in the regulation of hemocyte production. SGC, semi granular cells; GC, granular cells; TG, transglutaminase; ROS, reactive oxygen species; HPT cell, hematopoietic cells; HSC, hematopoietic stem cells (29, 45, 47, 75, 83–97).

The hematopoiesis involves several key molecular factors like Astakines (1 and 2), cytokine-like proteins that contain a prokineticin domain (PK), and playing the role of the growth factor hormone to stimulate the production and release of new hemocytes into circulation. Astakines acts through the reduction of the activity of transglutaminase (TGs) (**Figure 3**) (75, 83–89). TGs are Ca2+-dependent cross-linking enzymes with substrate is the type IV collagen, involved in coagulation and controlling hematopoiesis and extracellular matrix organization in crayfish (84, 88, 91, 92). TGs aims to inhibit stem cell differentiation and migration, which is closely dependent to ROS signaling (**Figure 3**). Astakine 1 was shown to stimulate differentiation along with SGC, through inducing the crustacean hematopoietic factor (CHF) to inhibits stem cell apoptosis and stimulates their differentiation and migration (75). Although, Astakine 2 most probably plays a role in the differentiation of GC (47, 75, 89). Astakine expression and release were reported to be induced by a bacterial infection (LPS), fungal challenge, and tissue injury (84, 86, 87, 91). Moreover, crayfish’ hemocytes reported as to be precursor for new neuron formation, and renewal of the nervous system in adult crayfish (93–95). Also, β-thymosin proteins are known for their actin-binding activity, and have been reported to interfere with Astakine in inducing hemocytes production and migration from the HPT (**Figure 3**) (96). In this same context, it is important to highlight the role of transglutaminase and crustin, a cysteine-rich anti-microbial peptide, as RNA binding proteins with a down-regulation role in post-transcriptional regulations for Astakine 1 expression (47, 97) (**Figure 3**).

## Integration of the Crayfish’ Immune Components Into Functional Mechanisms

### Role of Hemocyte Sub-populations in Mounting an Encapsulation

A large parasite that cannot be phagocytosed nor engulfed by hemocytes triggers the recruitment of new hemocytes into the infection site to isolate the invader by what is known as encapsulation. Such a process is usually accompanied by the activation of the ProPO system and the formation of a melanin sheath around the pathogen, which should be digested by quinones resulting from PO cascading reactions. These aim to neutralize the pathogen and prevent it from disseminating the hemocel (16, 21, 26, 98). Overall, inflammation consists of several successive steps starting with the recognition step of pathogens and/or sensing tissue damages (16, 17, 28, 30), followed by the recruitment of immune cells to the infected/damaged site, where additional effector molecules, such as clotting proteins (e.g., peroxinectins and transglutaminase), would be generated and released by these cells to consolidate pathogen isolation (i.e., capsule formation) (4, 14, 26, 84, 85, 88, 92, 99, 100) (**Figure 3**). These activities may also initiate further recruitment of neo-formed hemocytes through the secretion of cytokine-like proteins (Astakine 1 and 2), which stimulate stem cell proliferation and new hemocyte maturation (83, 85). However, Persson et al. (14) demonstrated in an *in vitro* assessment that capsule formation is mainly assured by semi-granular cells that are recruited and flattened to form aggregates around different spherules. Such capsules later develop into densely packed layers, which is not the case with granular cells forming less stable structures (4). In that same report, hyaline cells are described as the type that never participates in any aggregate formation (4). Other studies have reported that the hemocyte type involved in encapsulation depends on insect order (101). Dubovskiy et al. (101) stated that granular cells are the first to come in contact with and aggregate around the invader, therefore releasing clotting proteins to facilitate the termination of the process (101). In the same context, Jiravanichpaisal et al. (18) found that granular and semi-granular cells in crayfish are cooperatively involved in encapsulation, whereas no functional involvement has been allocated to hyaline cells (**Figure 1**) (18, 30, 32, 102).

### Prospective Role of the Extracellular Trap Cell Death Pathway (ETosis) in Encapsulation

Recent reports have highlighted the potential participation of HC in initiating encapsulation (32, 103). This finding has been demonstrated through the involvement of HC in implementing the extracellular trap cell death pathway (ETosis) in the crab *Carcinus maenas* (104, 105). In this context, it’s worth highlighting that ETosis is a phenomenon which was first described in mammals and which can be triggered by inflammatory cells like neutrophils and other mammalian innate immune cells (105, 106). Such a phenomenon entails an extracellular expulsion of chromatin out of the nucleus intending to entrap and kill foreign invaders like bacteria, fungi, viruses, and parasites. ETosis requires the involvement and production of reactive oxygen species (ROS) through the NADPH oxidase enzyme complex and myeloperoxidase (32, 105). The inflammatory condition is described to favor the initiation of the process by de-condensing the nucleus within the cell and then expelling the cloud-like mesh of chromatin into the extracellular environment in a controlled manner (104, 105). Such material was found to be accompanied by antimicrobial agents (as antimicrobial peptides from granules and histones with antimicrobial functions as well) (107). ETosis has been recently found in some arthropods, bivalves, and cnidarians (103–105, 107). ETosis in the crab has been proved to be caused by the prohemocytes (homolog of HC in crayfish) (32, 103). Interestingly, SGC also seems to have the ability to release chromatin (103). Most importantly, ETosis greatly facilitates the encapsulation process through the entrapment of pathogens by expelled chromatin, and aids in assembling the flattened layers of hemocytes to form a sort of sheath around the foreign entity using the externalized chromatin as a scaffold during the early stage of encapsulation (32, 104, 105). Peroxinectins and histones (mainly H2A) were also found to be greatly involved in the early stages of encapsulation (103, 105, 107). The ETosis pathway needs to be investigated in crayfish to be confirmed as a potential immune strategy that could be assured or at least require to be initiated through the involvement of HC.

### Sessile Hemocytes—Potential Role in the Immune Response

In contrast to circulating hemocytes in invertebrates, which are referred to as free wandering cells in the hemolymph, sessile hemocytes, also known as fixed phagocytes, are hemocytes that can migrate, infiltrate and inhabit different tissues and organs, due to the open circulatory system of invertebrates (30, 45, 101, 108, 109). Therefore, sessile hemocytes can inhabit in, and sometimes even be fixed to, different tissues (e.g., as in the central channel of the gills, arterioles, and hepatopancreas) (18, 110). Recently, the involvement of such cells in determining a proper immune response has been extensively studied, raising a putative role in immune defenses (108, 109). Amongst others, sessile hemocytes have been described to be involved in phagocytic activities aiming to clear foreign materials and substances from the hemocoel, and to accumulate debris within their giant vacuoles in some organs like the gills, and from the epithelium of the hepatopancreas (18, 30, 45, 110). More interestingly, a potential role of replenishment for circulating hemocytes, following immune challenge, has been suggested as a putative strategy *via* a third route rather than hematopoiesis (30, 45). Otherwise, still to be confirmed as a role of sessile hemocytes is that of signal delivery following pathogen recognition (through PRRs). This assumption has been corroborated by the fact that fixed hemocytes have resulted from the circulating hemocytes’ ability to migrate, attach and penetrate the basement membrane by extravasations into infection sites, even before the pathogen enters, as reviewed in Butt et al. (102). Recent studies have aimed at investigating the immune functions of sessile hemocytes from three different organs of Kuruma shrimp and have demonstrated the fact that such hemocytes differentially expressed immune-related genes compared to circulating hemocytes (108, 109). From the above, it’s highly important to note the ability of “peripheral” hemocytes of the eastern oyster *Crassostrea virginica* (described as pallial hemocytes; compared to those designated as sessile hemocytes in crustaceans) to ensure exchanges with circulatory hemocytes (111, 112). Such exchanges have been described as bi-directional transepithelial migrations, which proved to regulate the pathogenesis of its obligate parasite *Perkinsus marinus* (111, 112). Interestingly, it has been demonstrated that such peripheral hemocytes share common general characteristics with circulating ones but also display some differences in their cell surface phenotypes (112–114). Overall, reporting the characteristics and role of sessile hemocytes in crayfish defense strategies would help in improving the understanding of the crayfish immune response.

## Hyaline Cells and Neoplasia-Interference With the Immune Response

Since the development of the hemocyte’ classification system into sub-populations in crayfish, there has been much debate about the function of hyaline cells (HC), in spite of their very little shown phagocytic activity (5, 26, 43, 45, 46, 53, 82). Recent studies have suggested that HC are in fact free wandering prohemocytes that might be released into circulation only under certain circumstances. This hypothesis has led to much debate about HC’s identity, which is described as having hematopoietic cells’ characteristics’ (undifferentiated, expressing PCNA) (43, 45, 47, 76). This, in the light of the paradigm that clustering cell proliferation (excessive divisions), as can be exclusively occurs in the hematopoietic tissue, have strongly increased controversy over the identity of HC (32, 46, 52, 53, 85, 103), could be explained by the possible triggering of a neoplastic disease (e.g., lymphoma-like disease or hemic neoplasia) (32, 43, 45). However, chronic inflammatory conditions like excessive release of Astakine1 (role of growth factor hormone), besides the recorded involvement of PDGV-factor within signaling processes, which may stimulate an angiogenesis profile, favoring appearance of neoplastic features (83, 115). It is worth noting that cells with peculiar shapes that mark the initiation of neoplasia and occurrence in crustaceans, have been already reported, which might be assumed as a potential process explaining the hemocytes’ loss of immune capacity despite the recovery recorded in terms of number (i.e., increased THC after infections/challenges within different scenarios) (116).

Unfortunately, neoplasia in crayfish, and in crustaceans in general, hasn’t been studied much. The scarcity of information on tumors in crustaceans has been attributed to the lack of accurate descriptions by experienced crustacean pathologists, and to the absence in most reported cases of histopathological examinations (116–119). Neoplastic features mostly exhibit nodules containing numerous anaplastic and hypertrophied/giant cells, with either one large nucleus or several small nuclei. At the tissue level, possible tissue lesions presenting structural disorganization and high amounts of undifferentiated pleomorphic cells with hypertrophied nuclei and prominent nucleoli, in addition to bizarre multipolar mitotic figures have been reported (116–119). Despite the few documented case studies reporting crustacean neoplasia, a lack of accurate assessments has also been attributed to researchers’ backgrounds, having only previously reported some cases of neoplasia in assessments that were primarily concerned with other topics.

In this same context, it’s worth noting that studying the immune capacity of hemocytes should be considered while checking their expression of p53/63 and heat shock proteins (HSPs), such as HSP70 and HSP90, as biomarkers of neoplastic transformations.

## Molecular Components of Crayfish Immunity

### Pattern Recognition Receptors (PRRs)

**Figure 4:**
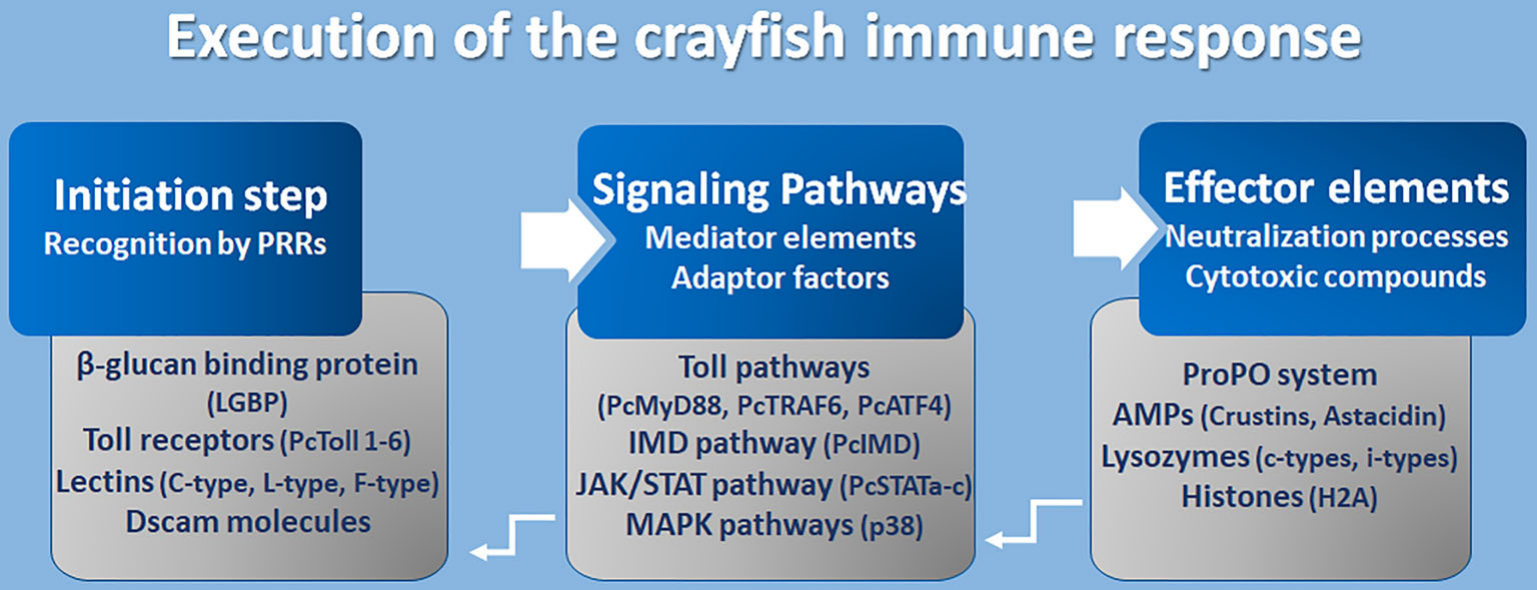
Execution of crayfish immune response. Coordination of the immune response at molecular level (8, 16, 17, 97, 106, 120–141).

So far it has been demonstrated that the activation of an effective, immune response requires the cooperative processing of molecular components in three mechanistic levels, which are the initiation process, response propagation through signal networking, and finally, the stimulation of effector molecules to neutralize pathogens (**Figure 4**).

However, the initiation step constitutes an accurate recognition of the pathogens or their associated cell walls (PAMPs like LPS and β-glucans) by the receptors of cells/epithelium on the external surfaces, enriched with components of innate immunity (PRRs). Many PRRs have been identified so far in freshwater crayfish such as toll-like receptors, β-glucan binding proteins (LGBP), lectins, integrins, and Down syndrome cell adhesion molecules (Dscams). Fortunately, the TLRs are one of the well-conserved families of PRRs that perform a recognition role of diverse classes of pathogens including fungi, bacteria, and viruses. Several Toll receptors and related proteins have been identified in different freshwater crayfish, for example, Toll receptor 3 from *A. astacus* (120), PcToll1, 2, 3, 4, 5, and 6 from *Procambarus clarkii* (121–127). Crayfish PRRs represent lectins as an important class of molecules with a role of non-self-recognition, however, lectins are proteins that bind to sugar using a characteristics carbohydrate recognition domain (CRD). In fact, among the 13 animal classes of lectins identified (according to their structural characteristics), crayfish lectins were reported to belong to at least three structural groups. Pc-Lec2 & 3 from *P. clarkii* as C-type lectins (126, 128), PcL-lectin from *P. clarkii* as L-type lectins (129), and PlFLP1 & 2 from *Pacifastacus leniusculus* as Ficolin-like lectins (130). Because of their role in activating the ProPO system, LGBP are the most studied type of PRRs and have been identified to belong to the typical β-1,3-glucanase related protein (BGRP) family that participates in the recognition of a wide range of pathogens, including bacteria and fungi like the *A. astaci*, the causative agent of the plague disease in crayfish [further details are found in (126)]. Additionally, the Down syndrome cell adhesion molecules (Dscam), the largest Ig superfamily (IgSF) members, have been identified in arthropods including crustaceans (131). It’s interesting to highlight that Dscam can produce thousands of isoforms (142). Dscam, presents membrane-bound and soluble forms (mDscam and sDscam), which are distinguished only by the presence/absence of transmembrane and cytoplasmic tail regions. Studies in the Chinese mitten crab *Eriocheir sinensis*, demonstrated that alternative splicing of Dscam can generate pathogen-specific isoforms with diverse epitopes in response to immune challenge, that potentially be able to recognize a broad spectrum of ligands. In fact, Li et al. (142) demonstrated that the mRNA and protein expressions of EsDscam were significantly upregulated after bacterial challenge, leading to produce isoforms found to bind specifically with the original bacteria to facilitate efficient clearance *via* phagocytosis by hemocytes. Li et al. (143) have elucidated that pathogen-induced particular soluble Dscam isoforms specifically binding with the original bacteria *via* epitope II, and then interact with membrane-bound Dscam that share the same extracellular regions *via* epitope I to promote phagocytosis. The cascading signal transduction mechanisms that activate post-Dscam binding pathogens remain not fully characterized. Dscams have been recorded in freshwater crayfish such as *Cherax quadricarinatus* and *P. leniusculus*, and have been proposed as key candidates for a somatically diversified receptor system speculated to be a hypervariable pattern-recognition receptor in crayfish immunity, playing the role of the cell surface receptor that triggers phagocytosis and opsonins due to the detection of soluble Dscam isoforms in crustacean hemolymphs (131, 132). Such findings have highlighted the putative involvement of Dscams as immunological effectors that promote immune memory through their ability to produce variable Ig domain combinations to promote the specificity of recognition and binding to a variety of pathogens through such splicing capacity (126, 144).

### Mediators and Signaling Adaptors

A successful recognition step conducted by PRR members activates several downstream reactions aiming to propagate the immune response signals. Such networking is assured through a multitude of intermediate factors and mediator molecules of the Toll pathway, immune deficiency (IMD) pathway, Janus kinase-signal transducer activator of transcription (JAK/STAT) pathways, Mitogen-activated protein kinases (MAPKs) pathways, and the cytokines-like network. Many transcripts that have been assigned to the Toll pathways have been recorded for *A. astacus* (120). Up to now, many Toll intermediate homologs and signaling pathway components, such as those of the TLR4 signaling pathway (the cytosolic adaptor PcMyD88, tumor necrosis factor 6 (PcTRAF6), and PcATF4) have been identified in the red swamp crayfish *P. clarkii* (124, 133). However, some other key components which are necessary in TLR4 signaling, such as IRAK4, IRAK1, TRAF6, TAK1, and IRB, or their corresponding counterparts, remain unidentified in crustaceans (126). Besides, some other intermediate molecules belonging to other signaling pathways like the IMD pathway, the JAK/STAT and MAPK pathways, have been described in studies elucidating immunity against Gram-negative bacteria and triggering defenses through peptidoglycan (PG) receptors, and in response to white spot syndrome virus (WSSV) in *P. clarkii* (117, 126). The WSSV challenge modulates the PcIMD expression. The IMD pathway, which has a conserved function for regulating antimicrobial peptides’ (AMP) expression in crayfish against Gram-negative bacteria, has also been reported to be involved in regulating the expression of crustins and lysozyme genes against WSSV (133). In addition, three different cDNA isoforms designated as PcSTATa, PcSTATb, and PcSTATc, belonging to the JAK/STAT signaling pathway, have proved to play a crucial role against WSSV in the red swamp crayfish *Procambarus clarkii* by modulating the expression levels of anti-lipopolysaccharide factors (PcALF1, PcALF2, PcALF3, PcALF4, PcALF5, and PcALF6) and crustin (PcCrus1, PcCrus2, PcCrus3, and PcCrus4) (134). Furthermore, the involvement of p38-MAPKinase, belonging to the serine/threonine protein kinases (MAPKs) (e.g., ERKs, JNKs, and p38s), in triggering the immune response in *P. clarkii* as results of exposure to cadmium has been demonstrated. As such, MAPKs could be irritated by various extracellular stimulations (e.g., inflammatory cytokines, pathogen infections, and environmental stress) (135).

### The Effector Elements of Immunity

Immune components leading to the implementation of functional defenses against pathogens, like the ProPO system, and their related activation molecules such as serine proteases and proteases inhibitors, together with their intermediate regulators, have been thoroughly investigated (8, 24, 136). The ProPO system activates the copper-containing enzyme phenoloxidase (PO) to oxidize phenols into quinones, which in turn are catalyzed into melanin. This process was found to be involved in wound healing and encapsulation as part of antifungal defenses [further details can be found in (8, 21, 27)].

AMPs are widely distributed small cationic polypeptides that exert an anti-pathogenic effect on a broad spectrum of pathogens. Crustacean AMPs can generally be classified into four groups according to their amino acid compositions and structural properties (106, 127) (1): single-domain amphipathic linear α-helical structure AMPs, including astacidins which are proline-rich peptides like astacidin 2 from *P. leniusculus* (137), and Pc-astacidin from *P. clarkii* (127) (2); single-domain peptides enriched with cysteine residues that are able to form at least one disulfide bridge between the B-sheets (3); AMPs with multi-domain linear α-helical peptides enriched with certain amino acids, generally proline and/or glycine residues, including crustins, characterized by their acidic protein (WAP) domain at C-terminus, such as Pl-crustin 1 & 2 in *P. leniusculus*, and Pc-crustin 4 in *P. clarkii* (138, 139), and procambarins, the glycine-rich peptide described in *P. clarkia* (140); and (4) unconventional AMPs including proteins/protein-fragments with antimicrobial functions like H2A histones and anti-lipopolysaccharide factors (ALFs) (reviewed in 121, 126). Lastly, at least two types of invertebrate (i-types) lysozymes, Pclysi 1 and 2, have been identified in *P. clarkii* (141). However, all lysozyme types described in crustaceans are of the chicken type (C-type) (126). Lysozymes are important factors with antimicrobial functions defined by their muramidase activity, hydrolyzing β-1,4-glycosidic linkage between N-acetylmuramic acid and N-acetylglucosamine, thus causing the lysis of peptidoglycan bacterial walls.

## Forward-Looking Summary of Developments in the Field

This review presents an extensive and up-to-date knowledge of crayfish immunity molecular components aiming to advance our understanding the defense mechanisms to evoke the old dilemma of plague disease, to enhance the resistance of endangered native European crayfishes. Unfortunately, early preventive strategies, like boosting crayfish immune responses, have not been fairly explored. Assessments of crayfish’s immune responses should be considered in light of the recent updates on signaling pathways and processes that were found to be involved in different anti-pathogenic defenses. It is also interesting to highlight what is described as host-pathogen interactions as an underlying factor influencing the adjustment of an effective immune response. However, a recent study conducted by Zheng et al. (145) demonstrated the involvement of the Toll pathways in the early detection of fungal infection, even before the pathogen penetrates the host cuticle. The *Locusta migratoria manilensis* can activate the Toll signaling pathway by upregulating the Spaetzle and Cactus members of the upstream PRRs of the Toll pathway following a challenge by β-1, 3-glucan (laminarin) from the cell wall of the fungus *Metarhizium acridum* when applied onto the host cuticle, even before fungal penetration (145). Such findings pave the way for further investigations taking into account the already described different signaling pathways in crayfish to increase our understanding of host-pathogen interactions in resolving the dilemma of crayfish plague disease. Yet, this aim could be more widen with integrative assessments taking into account the integration of the immune components into functional mechanisms. In doing so, understanding the importance of cellular versus humoral defenses in crayfish immune response is timely needed. This implies that greater emphasis needs to be placed on understanding the molecular mechanisms underlying cellular involvement in innate immune defenses of susceptible crayfish species. Such, may constitute a basis for wider assessments to overcome current limitations in forming immune priming strategies against *A. astaci* and to enhance conservation efforts of the endangered species. In this context, prospective acquirement of a specific immune response, named immunological memory of innate immunity, has been evidenced in many invertebrates, including crustaceans. However, deciphering the mechanisms of such memory is yet to be fully investigated. The main proposed underpinning molecular mechanistic is epigenetic re-programming or modification on gene expression due to different factors other than DNA mutations, which also remain elusive. To this end, an overreaching goal aiming to survey the modifications in the molecular mechanisms underlying the immunity of European freshwater crayfish, by exploring a potential transgenerational transfer of resistance traits against *A. astaci*, may consolidate breeding programs and crayfish production in captivity, thus enhancing European crayfish conservation.

## Author Contributions

The author confirms being the sole contributor of this work and has approved it for publication.

## Conflict of Interest

The author declares that the research was conducted in the absence of any commercial or financial relationships that could be construed as a potential conflict of interest.
